# A Fragile Image Watermarking Scheme in DWT Domain Using Chaotic Sequences and Error-Correcting Codes

**DOI:** 10.3390/e25030508

**Published:** 2023-03-16

**Authors:** Andy M. Ramos, José A. P. Artiles, Daniel P. B. Chaves, Cecilio Pimentel

**Affiliations:** Department of Electronics and Systems, Federal University of Pernambuco, Recife 50740-550, Brazil; andy.ramos@ufpe.br (A.M.R.); japalepg@gmail.com (J.A.P.A.); daniel.chaves@ufpe.br (D.P.B.C.)

**Keywords:** authentication, chaotic maps, error-correcting codes, discrete wavelet transform, image watermarking, tamper detection, security

## Abstract

With the rapid development of digital signal processing tools, image contents can be easily manipulated or maliciously tampered with. Fragile watermarking has been largely used for content authentication purposes. This article presents a new proposal for image fragile watermarking algorithms for tamper detection and image recovery. The watermarked bits are obtained from the parity bits of an error-correcting code whose message is formed from a binary chaotic sequence (generated from a secret key known to all legitimate users) and from bits of the original image. Part of the codeword (the chaotic bits) is perfectly known to these users during the extraction phase, adding security and robustness to the watermarking method. The watermarked bits are inserted at specific sub-bands of the discrete wavelet transform of the original image and are used as authentication bits for the tamper detection process. The imperceptibility, detection, and recovery of this algorithm are tested for various common attacks over digital images. The proposed algorithm is analyzed for both grayscale and colored images. Comparison results reveal that the proposed technique performs better than some existing methods.

## 1. Introduction

Digital watermarking is a technique of hiding information in multimedia data in such a way that the distortion due to watermarking is almost perceptually negligible [1]. Watermarking can serve a variety of purposes including copyright protection and data authentication. Image watermarking is the process of embedding binary information (called watermarked bits) into an original image, generating a watermarked image. In a self-embedding watermarking scheme, the watermarked bits are generated from the original image. The extraction process is called blind when it does not require knowledge of either the original image or the watermarked bits.

In general, image watermarking techniques can be categorized as robust, semi-fragile and fragile [2,3,4]. Robust watermarks are designed to survive image processing operations, such as scaling, cropping, filtering, and compression [5,6,7,8,9], and are usually used for copyright protection to declare ownership. Fragile watermarking is designed for detecting any modification of the watermarked image (tamper detection) and for recovering the tampered areas (image recovery) [2]. Semi-fragile schemes are designed for tamper detection and image recovery and are robust against some image processing operations. Their main disadvantage is a reduced recovering rate when compared to that achieved by fragile schemes. Fragile and semi-fragile watermarking schemes are mainly used for authentication purposes.

This work focuses on image fragile watermarking. The primary purpose is to invisibly embed a binary image (called watermark image) into an original image, creating a watermarked image, and then extract the embedded information from the watermarked image at the destination. The watermark is fragile in the sense that it is designed to be easily altered if any changes are made to the watermarked image, hence providing a means of detecting tampering or unauthorized alterations. Fragile watermarking can therefore be applied to copyright protection, tamper detection, and authentication.

In many applications, tamper detection and localization alone are insufficient. The fragile watermarking with self-recovery capability cannot only identify the tampered regions, but also recover the altered image’s original content. At the destination, the authentication watermark is first extracted and applied to identify the authenticity of the received image. If the watermarked image is identified as tampered, the restoration technique is applied to the tampered parts.

In many image fragile watermarking schemes, the original image is divided into non-overlapping sub-blocks and the watermark embedded in each sub-block is composed of authentication bits and recovery bits [10,11,12,13,14,15,16,17]. The authentication bits are used for the purpose of tampering detection (the block is authenticated if the authentication bits are successfully retrieved). The tampered blocks are recovered by means of the recovered bits. The generation of the watermarked bits involves, in some cases, frequency-domain transforms, such as the discrete cosine transform (DCT) [15,18], and the discrete wavelet transform (DWT) [14,19,20,21].

The performance of an image watermarking scheme is analyzed with mutually exclusive parameters, including imperceptibility, capacity, and robustness against attacks. Trying to improve one of these parameters for a particular scheme usually deteriorates the others [1]. Several embedding schemes are based on the least significant bit (LSB) method [11,12,13,16,19,22], since it provides a good trade-off among these performance metrics.

Chaotic maps are commonly used to add security to image watermarking schemes [13,14,22,23,24,25]. These maps are characterized by their sensitivity to the initial conditions and pseudo-random behavior, despite being deterministic, resulting in noise-like signals [14,26,27]. Applications of these maps include scrambling the original image [13,14,22] and selecting sub-blocks to embed the watermark [14,22]. To support severe distortion imposed on the watermarked image, error-correction codes can also be applied [18,28,29].

In this work, we propose a new self-embedding fragile watermarking algorithm for image tamper localization and recovery using chaotic maps, DWT domain, and error-correcting codes. The bits embedded in the image are obtained from parity bits of an error-correcting code whose information sequence is formed by combining the watermarked bits with chaotic bits generated from a secret key. The distinguishing feature of the proposed extraction algorithm is that the error-correcting capability of the error-correction code is exclusively dedicated to recovering the watermarked bits, since part of the codeword (the chaotic bits) is known by the extraction algorithm, which provides high robustness to the proposed scheme. The DWT sub-bands are divided into non-overlapping 2×2 sub-blocks and two parity bits are embedded in each sub-block. These bits are used as authentication bits for the tamper detection process. A parameter controls the trade-off between imperceptibility and robustness. After locating the tampered area, in the process of recovering the damaged area, the parity bits and chaotic sequences are used to estimate the recovery bits. We investigate the trade-off between the imperceptibility of the watermarking embedding and the tampering detection/recovering capability of the proposed algorithm and comparison results reveal that it performs better than many existing fragile watermarking schemes.

The rest of this article is organized into five sections. Section 2 presents a brief review of the chaotic maps, the class of error-correcting codes considered in this work, and DWT. The proposed algorithm for grayscale images is detailed in Section 3. It is also discussed the watermark extraction, tamper detection, and the image recovery strategy. Some metrics commonly used for assessing the imperceptibility, detection, and recovery capability of a fragile watermarking algorithm are discussed in Section 4. Results with performance comparisons are presented in Section 5 for grayscale images, and in Section 6 for colored images. Conclusion remarks are outlined in Section 7.

### Related Works

In this section, we briefly review several fragile watermarking schemes proposed in the literature.

Haghighi et al. [22] proposed a fragile blind watermarking scheme, based on lifting wavelet transform (LWT) and genetic algorithms. In this scheme, four digests are generated based on LWT and halftoning techniques. Each digest is separately scrambled using a chaotic map. The authentication bits for each 2×2 non-overlapping sub-block are calculated based on a relation of pixels. The watermarked bits are formed from a combination of digests and authentication bits and are embedded using the LSB technique. A genetic algorithm is employed to optimize the difference between the original and the watermarked values of each sub-block.

Barani et al. [23] proposed a digital image tamper detection algorithm based on the integer wavelet transform (IWT) and singular value decomposition (SVD). A SVD is performed in each 2×2 sub-block of the scrambled original image. The combination of the *U* matrix of the SVD of each sub-block and a sequence generated by a 3D quantum chaotic map forms an authentication sequence that is inserted into the IWT coefficients. A scheme that combines SVD and chaotic maps is proposed in the Ref. [25].

In the image fragile watermark scheme proposed in the Ref. [14], the original image is divided into 4×4 non-overlapping sub-blocks and the authentication and the recovery bits are both generated by using the DWT. The authentication bits are generated from the low-frequency sub-band of each sub-block, and the recovery bits are produced from high-frequency sub-bands. The chaotic Arnold’s cat map scrambles image sub-blocks in order to break their interdependence.

In the Ref. [30], Qin et al. proposed a self-embedding fragile watermarking scheme using vector quantization (VQ) and index sharing. The watermarked bits are composed of hash bits for tampering localization and reference bits for content recovery. The proposed scheme can locate tampered regions via VQ index reconstruction. Qin et al. [11] developed a self-embedding fragile watermarking based on reference data interleaving mechanism. This scheme utilizes the most significant bit (MSB) layers to generate the interleaved reference bits that are embedded into the LSBs. The scheme proposed in the Ref. [16] embeds the watermarked bits generated by a permutation process within the two LSB of each sub-block. A bit-adjustment phase is subsequently applied to increase the quality of the watermarked image. In the Ref. [31], the original image is divided into non-overlapping sub-blocks of 2×2 pixels, called small blocks, and each 4×4 small block is grouped as a large block. The watermarked bits containing authentication information and recovery information are embedded into the LSB.

In the Ref. [15], authentication data is generated for each 8×8 sub-block using the DCT. A block dependency is established using part of the authentication data of a distant block. Such sub-block dependency provides tamper detection and enables localization of tampered regions. A recovery technique based on unsupervised machine learning is proposed. The scheme presented in the Ref. [32] is also based on the DCT. Two authentication bits and ten recovery bits are generated from the five MSB of each sub-block. The authentication bits of each sub-block are embedded into the three LSB.

The algorithm proposed in the Ref. [12] consists of an overlapping block-wise mechanism for tampering detection and a pixel-wise mechanism for image recovery. Reference bits are derived from the mean value of each sub-block and are dispersedly hidden into 1 or 2 LSB according to two different embedding modes. Authentication bits are hidden into adaptive LSB layers of the central pixel for each block. After detecting tampered blocks and reconstructing mean-value bits, the original pixels are recovered using a pixel-wise approach with the help of different neighboring overlapping blocks. According to [17], two different types of detection processes, pixel-wise and block-wise processes, are suggested in order to locate and restore the tampered locations. The authentication data are created per pixel in the pixel-wise procedure while they are formed per block in the block-wise process. As a result, the block-wise method tunes the length of authentication data to the size of each block.

Peng et al. proposed in the Ref. [10] an algorithm based on reversible data hiding. The authentication and recovery bits are embedded into two identical original images. Secret information is embedded into one image while distortion information is embedded into the other one. In the Ref. [13], Sreenivas et al. proposed an image tamper localization scheme in which authentication bits of a 2×2 image sub-block are generated using chaotic maps. For each sub-block, two distinct sets of recovery bits are generated and embedded in the LSBs of two randomly chosen blocks. In the Ref. [33], a secret key based on pseudo-random binary sequences is used as a fragile watermark for tamper detection. The watermarked bits are embedded using a LSB process in a 9-base notation structure.

Li et al. [34] proposed an image tampering detection and a self-recovery method based on the Gauss–Jordan Elimination. A technique called Improved Check Bits Generation (ICBG) generates the check bits for tamper detection. The Morphological Processing-Based Enhancement (MPBE) is developed to improve the accuracy of tampering detection.

The scheme proposed in the Ref. [19] used two watermarks that combines spatial and transform domains to enhance the watermarking robustness, authentication and recovery performance. A robust watermarking is embedded into different DWT sub-bands, while a fragile one is embedded using the LSB approach. An image authentication system that combines DWT and convolutional neural networks (CNN) is proposed in the Ref. [20]. The watermark information is embedded into the DWT coefficients, and the CNN is employed to recover tampered areas. The combination of DCT and CNN is proposed in the Ref. [35].

Multiple median watermarking is a technique for image tamper region recognition and self-recovery proposed in the Ref. [36]. Four smaller versions of the cover image are hidden into the 4-LSB, which are determined by four pseudo-random codes. These copies can be used to identify the area of the altered image that has been tampered with.

## 2. Preliminaries

### 2.1. BCH Code

A well-known and powerful tool to enhance the robustness of a watermarking scheme is the use of error-correcting codes which permits to correct errors induced by a given attack [28,37,38]. This work employs the binary Bose, Chaudhuri, and Hocquenghem (BCH) code [39] over the Galois field GF(*q*) with codewords of length $n\;=\;q^{m}-1$ (where *m* is an integer), *k* information bits, n−k parity bits, and error correction capability *t* bits. It is denoted by BCH (n,k,t). This code is completely specified by its generator polynomial $g\left(x\right)\;=\;1+g_{1}x+\cdots +g_{n-k-1}x^{n-k-1}+x^{n-k}$, where $g_{i}\in \mathrm{GF}\left(q\right)$. The degree of g(x) is equal to the number of parity bits of the code. Let ξ be a primitive element of GF$\left(2^{4}\right)$, and let $m_{i}\left(x\right)$ be the minimal polynomial of $\xi^{i}$ in GF$\left(2^{4}\right)$, the i-th power of ξ. The generator polynomial g(x) is obtained from the least common multiple of the minimum polynomials $g\left(x\right)\;=\;\mathrm{LCM}(m_{1}\left(x\right),m_{2}\left(x\right),\ldots ,m_{d_{m}-1}\left(x\right))$, where $d_{m}$ is the code minimum distance and satisfies $d_{m}\geq 2t+1$.

A polynomial representation c(x) of a codeword $c\;=\;(c_{0},\cdots ,c_{n-1})$ is of the form $c\left(x\right)\;=\;c_{0}+c_{1}x+\cdots +c_{n-1}x^{n-1}$. The encoder operation can be expressed in the polynomial form c(x)=g(x)u(x), where u(x) is the information message to be encoded and the operations with polynomials follow the operations rules defined over the field. There are several techniques to decoding BCH codewords, it is worth mentioning the Berlekamp–Massey (BM) algorithm, an algebraic decoding algorithm for the BCH code. For the one-bit error correction code considered here, the generator polynomial is $g\left(x\right)\;=\;x^{4}+x+1$, for q=2, m=4, with n=15, and k=11 information bits, which has 4 parity bits and t=1, represented as BCH(15,11,1). We also use a BCH code with t=2, BCH(31,21,2), with 10 parity bits and generator polynomial $g\left(x\right)\;=\;x^{10}+x^{9}+x^{8}+x^{6}+x^{5}+x^{3}+1$. The use of both codes permits analyzing the impact of the code parameters on the watermarking algorithm.

In the proposed algorithm, the *k* information bits are split into 2 parts, one containing bits from the watermark image and the other a chaotic sequence generated from a secret key shared by legitimate users. The encoder operation finds the parity bits that are embedded into the original image, generating the watermarked image. The watermark extraction algorithm estimates the parity bits from a possibly modified watermarked image. A legitimate user generates the chaotic sequence and concatenates it with the retrieved parity bits. A decoding procedure estimates the watermarked bits forming the retrieved codeword (the concatenation of the estimated watermarking bits, chaotic sequence, and the estimated parity bits). It is worth highlighting that if the retrieved codeword contains errors (which occur if the errors introduced by an attack are beyond the error correction capability), these are spread out over all portions of the codeword and do not necessarily concentrate in the portion of the information sequence where the watermarked bits are located. However, the positions of the chaotic bits are known in advance, since this information is shared by all legitimate users. This allows for concentration of the code’s correctness capability on the portion of the codeword that contains the relevant information, the parity and watermarked bits.

### 2.2. Chaotic Maps

The behavior of unidimensional chaotic maps is observed through a discrete time series ${\left\{x_{i}\right\}}_{i=0}^{\infty }$, and can be obtained by iterating a nonlinear and non-invertible function f(x), over an initial condition $x_{0}$, as follows [40]

(1) $$x_{n}\;=\;f\left(x_{n-1}\right),\;n=1,2,3,\ldots$$

**Figure 1:**
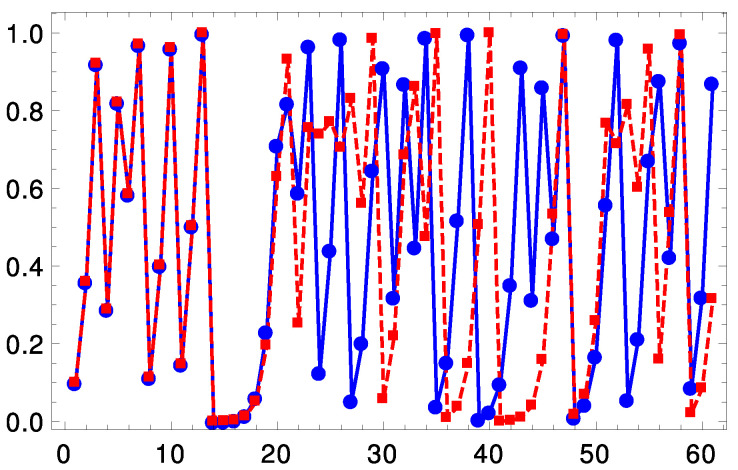
Two different orbits using (1) for the logistic map with r=4. The initial conditions are $x_{0}\;=\;0.1$ and $x_{0}^{\prime }\;=\;0.100001$.

The sequence ${\left\{x_{n}\right\}}_{n=0}^{\infty }=\left\{x_{0},f\left(x_{0}\right),f\left(f\left(x_{0}\right)\right),\ldots \right\}$ is called an orbit of f(x). The value of $x_{0}$ is obtained from the secret key and we choose to discard the first 100 samples of an orbit due to the transient behavior (the orbits of a good chaotic map diverge after few iteration and this discard operation is not mandatory). Examples of chaotic maps include the cubic map (MC) $f\left(x\right)\;=\;4x^{3}-3x$, and the logistic map (ML) f(x)=rx(1−x), where *r* is a control parameter. Chaotic maps are deeply sensitive to the initial condition, meaning that arbitrarily close initial points separate exponentially fast. Figure 1 shows two orbits of the logistic map with r=4 generated by two initial conditions separated by $10^{-6}$. These assume a distinct dynamical behavior after a few iterations.

The balanced binary sequence $\left\{z_{n}\right\}$, henceforth denoted by the chaotic binary sequence, is generated from $\left\{x_{n}\right\}$ from a partition of the map domain into two regions $R_{0}$ and $R_{1}$ satisfying $\mathrm{Pr}\left(x_{n}\in R_{0}\right)=\mathrm{Pr}\left(x_{n}\in R_{1}\right)=1/2$, and such that, if $x_{n}\in R_{0}$ then $z_{n}\;=\;0$, or if $x_{n}\in R_{1}$ then $z_{n}\;=\;1$. There are several methods to discretize chaotic samples (see, for example, [41,42,43]) that may generate binary sequences with better random properties, but this topic is not explored in this work.

### 2.3. Discrete Wavelet Transform

The basis functions of the DWT are generated from a basic wavelet function, through translations and dilations. These functions allow reconstructing the original signal through the inverse discrete wavelet transform (IDWT). There are many types of wavelet functions, including Haar [44,45], Daubechies [46], Symlets [47], Coifflets [48,49]. Due to its low computing requirements, the Haar transformation has been used primarily for image processing and pattern recognition and is adopted in this work.

**Figure 2:**
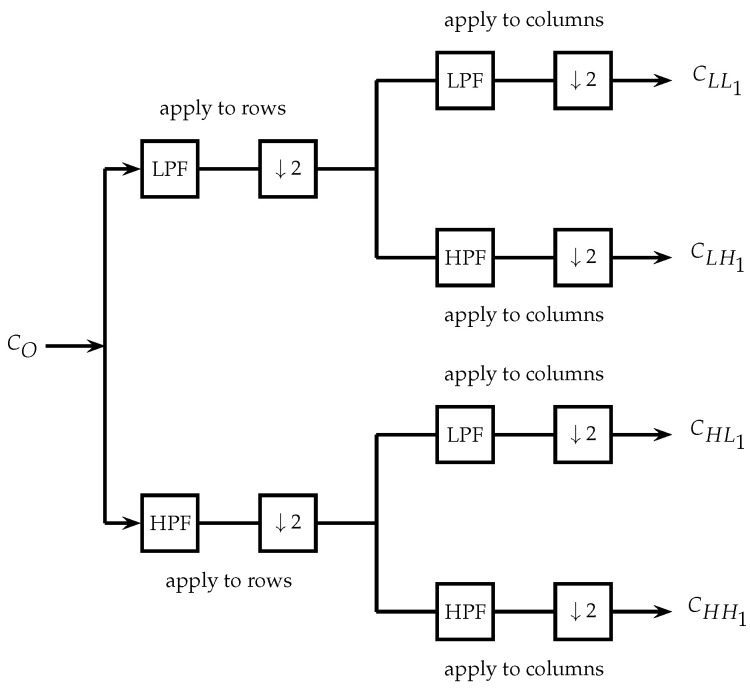
Wavelet decomposition scheme in two dimensions.

Mallat [50] proposed an algorithm based on a decomposition following a pyramid model, in which the image size decreases in each decomposition level. Figure 2 shows the first decomposition level applied to an image $C_{O}$ of size M×N, obtaining four output images $C_{LL_{1}},C_{LH_{1}},C_{HL_{1}},C_{HH_{1}}$ of size M/2×N/2. At the end of each filtering operation, the output signal is down-sampled by two (↓2). The image $C_{LL_{1}}$ is obtained from the convolution of two low-pass filters applied first to the rows and then to the columns of $C_{O}$. The first level of detail $C_{LH_{1}}$ is obtained by applying a low-pass filter to the rows of $C_{O}$ and then a high-pass filter to its columns. Similarly, $C_{HL_{1}}$ and $C_{HH_{1}}$ are obtained. Applying this procedure again having as input the image approximation $C_{LL_{1}}$, we obtain the second decomposition level of the image $C_{O}$, resulting in the approximations $C_{LL_{2}}$ and the level of details $C_{LH_{2}}$, $C_{HL_{2}}$, $C_{HH_{2}}$, each one with size a quarter of the size of image $C_{O}$. Other decomposition levels are obtained using the same procedure.

## 3. The Proposed Algorithm

A new fragile watermarking algorithm for images as well as a strategy for tamper detection and recovering of the tampered areas are proposed in this section. Initially, we consider grayscale images. colored images are considered in Section 6.

The embedding algorithm $E^{\cdot }$ has as input an 8-bit grayscale original image $C_{O}$ of size M×N pixels and a key *K* which determines the initial condition $x_{0}$ of the chaotic sequence. The watermarked image $C_{W}$ is described as

(2) $$C_{W}=E\left(C_{O},K\right).$$

The input to the blind extraction algorithm $E^{-}\left(\cdot \right)$ is the watermarked image possibly corrupted by attacks, namely $C_{W}^{\prime }$, and a key *K*.

### 3.1. Watermark Embedding

Watermarked bits are embedded into the original image according to the following steps.

1. Generate a chaotic binary sequence $SC_{1}$ using the cubic chaotic map with the key *K*.
2. Apply the 2-level DWT decomposition to the original image $C_{O}$ obtaining the sub-bands $C_{LL_{2}}$, $C_{LH_{2}}$, $C_{HL_{2}}$, $C_{HH_{2}}$. The sub-bands $C_{LH_{2}}$ and $C_{HL_{2}}$ (each one of size M/4×N/4 pixels) are divided into sub-blocks of size 2×2, where the watermarked bits are embedded. There are $\frac{MN}{64}$ sub-blocks in each sub-band. Each sub-block is composed of the coefficients:
$c_{11}$$c_{12}$$c_{21}$$c_{22}$
3. Apply the 4-level DWT decomposition to the original image $C_{O}$. The image $C_{LL_{4}}$ has size M/16×N/16 pixels. Convert each byte of this image to a binary sequence $\ell_{4}$ of length $\frac{MN}{32}$ bits.
4. Construct the parity check sequence p of the BCH (15,11,1) code as follows. The 11 information bits are obtained by concatenating $k_{1}$ bits from $\ell_{4}$ and $k_{2}$ from the chaotic map ($SC_{1}$ sequence), where $k_{1}+k_{2}\;=\;11$. After encoding, a 15-bit codeword is obtained with 4 parity bits. After repeating this process for the entire $\ell_{4}$, a parity sequence of size $\frac{MN}{8k_{1}}$ is obtained. This sequence is considered as an image and is scrambled with the Arnold cat map. After scrambled, this sequence is divided into sub-sequences of length 2 bits, $p\;=\;\{p_{1},p_{2},\ldots ,p_{\frac{MN}{16k_{1}}}\}$, where $p_{i}=p_{i1},p_{i2}$. Each $p_{i}$ is embedded into the sub-blocks of $C_{LH_{2}}$ and $C_{HL_{2}}$.
5. In each 2×2 sub-block of $C_{LH_{2}}$ and $C_{HL_{2}}$ find the largest value ($v_{max1}$) and the second largest value ($v_{max2}$) of $c_{11},c_{12},c_{21},c_{22}$. Let $\alpha_{1}\;=\;v_{max1}-v_{max2}$. If $\alpha_{1}\leq \alpha$, where α is a fixed positive parameter for all sub-blocks, then $v_{max1}\leftarrow \;v_{max1}+\alpha$, otherwise $v_{max1}$ remains unchanged. The choice of α involves a trade-off between imperceptibility and robustness, as is discussed in the next sections. Each sub-sequence $p_{i}$ is embedded in each sub-block of each sub-band according the following rules (consider that $v_{max1}$ is in position $\left(i_{1},j_{1}\right)$ of the sub-block, $1\leq i_{1},j_{1}\leq 2$):
  - If $p_{i}\;=\;00$, then replace $c_{i_{1}j_{1}}$ by $c_{11}$ and $c_{11}$ by $v_{max1}$.
  - If $p_{i}\;=\;01$, then replace $c_{i_{1}j_{1}}$ by $c_{12}$ and $c_{12}$ by $v_{max1}$.
  - If $p_{i}obtain\;=\;10$, then replace $c_{i_{1}j_{1}}$ by $c_{21}$ and $c_{21}$ by $v_{max1}$.
  - If $p_{i}\;=\;11$, then replace $c_{i_{1}j_{1}}$ by $c_{22}$ and $c_{22}$ by $v_{max1}$.
6. Apply the two-level IDWT and obtain the watermarked image $C_{W}$.

**Figure 3:**
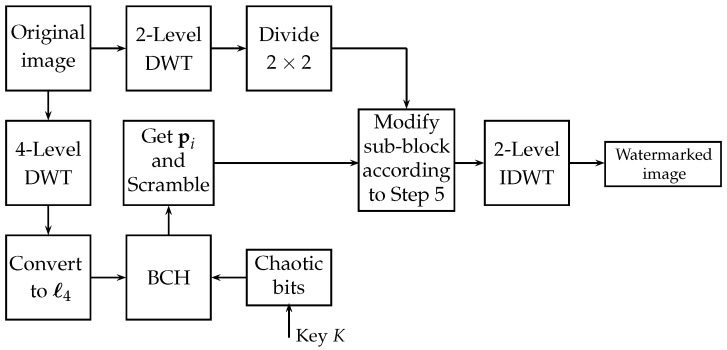
Block diagram of the proposed watermark embedding algorithm.

Since the number of sub-sequences $p_{i}$ is $\frac{MN}{16k_{1}}$ and the total number of sub-blocks is $\frac{MN}{32}$, we have $k_{1}=2$, and consequently $k_{2}\;=\;9$. Figure 3 shows the block diagram of the proposed embedded algorithm, called Proposed 1.

### 3.2. Watermark Extraction, Tamper Detection, and Image Recovery

The embedding of watermarked bits in $C_{O}$ allows detecting modifications (tamper detection) and to recover the original image (image recovery).

#### 3.2.1. Watermark Extraction

The extraction of parity sequence $\hat{p}$ from $C_{W}^{\prime }$ (possibly modified watermarked image) and from *K* is based on the following steps.

- Generate the chaotic binary sequence $SC_{1}$ from the key *K*.
- Calculate the two-level DWT of $C_{W}^{\prime }$ obtaining the sub-bands $C_{LH_{2}}$ and $C_{HL_{2}}$. Each sub-band is divided into sub-blocks of size 2×2.
- Find the highest value $v_{max}^{\prime }$ of each sub-block and its position. Decide the watermark information ${\hat{p}}_{i}$ as:
  - If $v_{max}^{\prime }$ is in the position (1,1), then ${\hat{p}}_{i}\;=\;00$.
  - If $v_{max}^{\prime }$ is in the position (1,2), then ${\hat{p}}_{i}\;=\;01$.
  - If $v_{max}^{\prime }$ is in the position (2,1), then ${\hat{p}}_{i}\;=\;10$.
  - If $v_{max}^{\prime }$ is in the position (2,2), then ${\hat{p}}_{i}\;=\;11$.
- The estimated parity sequence is unscrambled with $K_{1}$ and is divided into 4-bit sub-sequences, ${\hat{p}}_{j}\;=\;{\hat{p}}_{j1}\cdots {\hat{p}}_{j4}$, for $j\;=\;1,\ldots ,\frac{MN}{64}$.
- For each ${\hat{p}}_{j}$, the extraction algorithm knows $k_{2}=9$ chaotic bits of an 11-bit information sequence. There are four possible parity sequences, depending on the remaining $k_{1}=2$ information bits. An estimate of these bits is obtained from the smallest Hamming distance between ${\hat{p}}_{j}$ and these possible parity sequences. Then, concatenate the estimated $k_{1}$ bits, the $k_{2}$ the chaotic bits, and the four parity bits with the smallest Hamming distance to form a 15-bit word. This word is decoded using the BM algorithm, giving a new estimate of the $k_{1}$ bits of the sequence $\ell_{4}$ and ${\hat{p}}_{j}$.
- This procedure is repeated for each $j\;=\;1,\ldots ,\frac{MN}{64}$, obtaining two estimated sequences $\hat{p}$ and ${\hat{\ell }}_{4}$.

**Figure 4:**
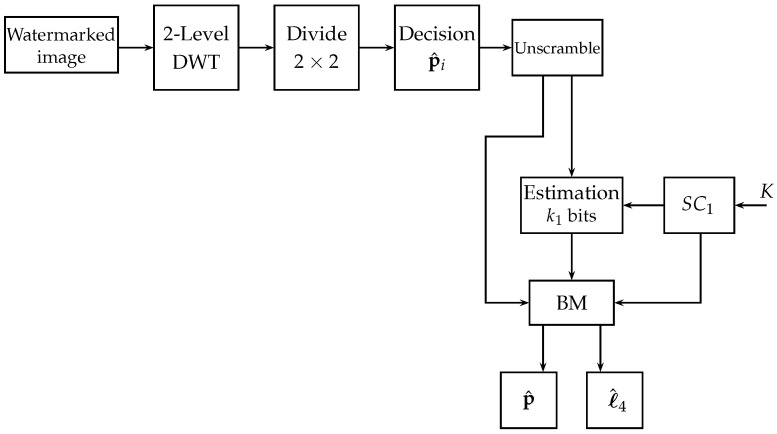
Block diagram of the proposed watermark extraction algorithm.

Figure 4 shows the block diagram of the proposed watermark extraction algorithm.

#### 3.2.2. Tamper Detection

**Figure 5:**
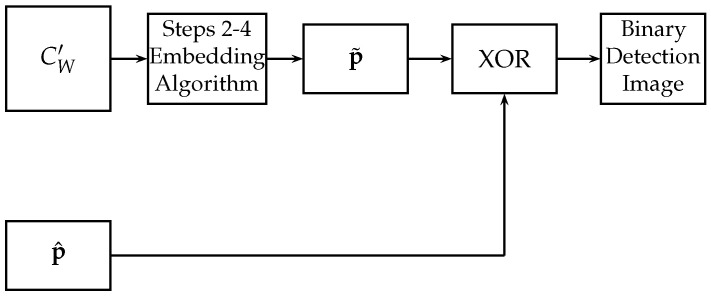
Block diagram of the proposed tamper detection algorithm.

The image $C_{W}^{\prime }$ is used to replicate Steps 2–4 of the embedding algorithm, obtaining a new binary sequence $\tilde{p}$ of length $\frac{MN}{8}$. In order to detect the tampered regions, a bitwise XOR operation is performed between the extracted watermark binary sequence $\hat{p}$ and the binary sequence $\tilde{p}$. The binary sequence resulting from this operation is organized in a binary image of size M/4×N/4 bits, which is called binary detection image. Figure 5 shows the block diagram of the proposed tamper detection algorithm.

#### 3.2.3. Image Recovery

**Figure 6:**
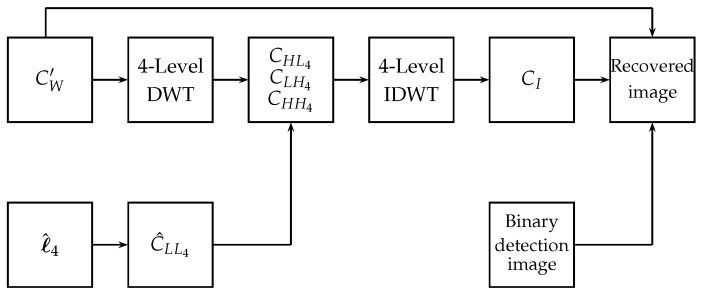
Block diagram of the proposed recovery algorithm.

After detecting if there is any modification in the watermarked image $C_{W}^{\prime }$, the next step is to recover the part of the image identified as tampered. In the recovering process, the first step is to calculate the details and sub-bands $C_{HH_{4}}$, $C_{HL_{4}}$ and $C_{LH_{4}}$ of the tampered image $C_{W}^{\prime }$. The binary sequence ${\hat{\ell }}_{4}$ is converted to the image ${\hat{C}}_{{LL}_{4}}$ of size M/16×N/16 pixels. An intermediate image $C_{I}$ is obtained from the 4-level IDWT of the image formed from ${\hat{C}}_{{LL}_{4}}$, $C_{HH_{4}}$, $C_{HL_{4}}$, and $C_{LH_{4}}$. The recovered image is constructed by replacing the pixels located at the detected tampered area of $C_{W}^{\prime }$ by the corresponding pixels of $C_{I}$. Figure 6 shows the block diagram of the proposed image recovery algorithm.

## 4. Imperceptibility, Detection, and Recovery Metrics

This section describes commonly used metrics for assessing the imperceptibility and robustness of image watermarking schemes.

### 4.1. Imperceptibility Metrics

The peak signal-to-noise ratio (PSNR) is a measure of watermark imperceptibility, expressed in units of decibels (dB). For 8-bit grayscale images with pixel values from 0 to 255, the PSNR is defined as

(3) $$\mathrm{PSNR}\;=\;10\log_{10}\left(\frac{255^{2}}{\mathrm{MSE}}\right)\;\left(\mathrm{dB}\right)$$

where the mean square error (MSE) for images of size M×N is

(4) $$\mathrm{MSE}\;=\;\frac{1}{M\times N}\sum_{i=1}^{M}\sum_{j=1}^{N}{\left(C_{O}\left(i,j\right)-C_{W}\left(i,j\right)\right)}^{2}.$$

The recovered PSNR, ${\mathrm{PSNR}}^{r}$, is calculated using (3) in which the MSE is obtained between the watermarked image and recovered image. The structural similarity index (SSIM) is another imperceptibility metric and is defined as

(5) $$\mathrm{SSIM}\;=\;\frac{\left(2\mu_{O}\mu_{W}+\gamma \right)\left(2\rho_{OW}+\beta \right)}{\left(\mu_{O}^{2}+\mu_{W}^{2}+\gamma \right)\left(\sigma_{O}^{2}\sigma_{W}^{2}+\beta \right)}$$

where $\mu_{O}$ and $\mu_{W}$ are the mean of the original and watermarked images, respectively, $\sigma_{O}^{2}$ and $\sigma_{W}^{2}$ are the variances of these images, $\rho_{OW}$ is the covariance between $C_{O}$ and $C_{W}$, γ and β are fixed constants, γ=2.55 and β=7.65.

### 4.2. Tampered Detection Metric

The performance of tamper detection is commonly measured in terms of the false positive rate (FPR) and false negative rate (FNR), defined as

(6) $$\begin{array}{ccc} \mathrm{FPR} & = & \frac{\mathrm{FP}}{\mathrm{TN}+\mathrm{FP}} \end{array}$$

(7) $$\begin{array}{ccc} \mathrm{FNR} & = & \frac{\mathrm{FN}}{\mathrm{FN}+\mathrm{TP}} \end{array}$$

where FP, FN, TP, TN are the false positive, false negative, true positive, and true negative, respectively. FP is the number of pixels that are non-tampered but are wrongly identified as tampered; FN is the number of pixels that are tampered but are incorrectly detected as non-tampered; TP is the number of pixels that are correctly identified as a tampered pixel, and TN is the number of pixels that are correctly identified as an untampered pixel. The lower FPR and FNR indicate a better performance of the tamper detection algorithm.

### 4.3. Watermark Image Attacks

Several attacks are performed on the watermarked image to check the behavior of the proposed algorithm, as described next.

- In the tamper attack, the pixels of a part of $C_{W}$ are changed to zero [15].
- The first kind of collage attack ($CA_{1}$) tampers the $C_{W}$ image by copying blocks of $C_{W}$ and inserting them into arbitrary positions in the same watermarked image [14,15].
- The second kind of collage attack ($CA_{2}$) modifies $C_{W}$ by combining portions of another watermarked image and preserving their relative spatial locations [14,15,17].
- In the normal tampering attack, some objects are added, deleted or modified on the watermarked image [22].
- The salt and pepper attack consists in adding this noise with density *d* to the $C_{W}$ image [17].
- The constant-average attack (CAA) [14,15] is able to tamper a set of blocks with a constant average intensity and create a counterfeit image. The average value for each block in the tampered area is calculated, and then the 6 MSBs of each pixel, within the block, are replaced by the 6 MSBs of the calculated average value [15].

**Figure 7:**
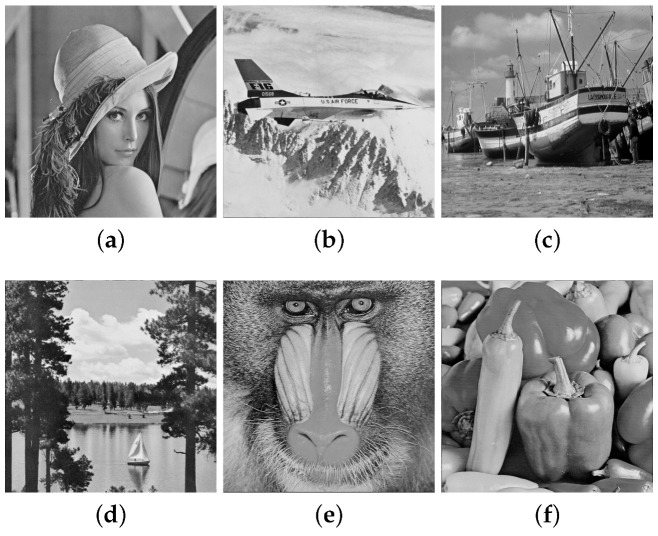
(**a**) Lena, (**b**) Airplane, (**c**) Boat, (**d**) Lake, (**e**) Baboon, (**f**) Pepper.

The performance analysis conducted in this chapter uses 141 original images from the USC-SIPI database (http://sipi.usc.edu/database, accessed on 10 March 2021). This database contains grayscale and colored images of distinct sizes. We resize and convert some images so that a new database contains 8-bit grayscale images of size 512×512 pixels. Figure 7 shows some examples of images used in this work.

## 5. Results for Grayscale Images

**Figure 8:**
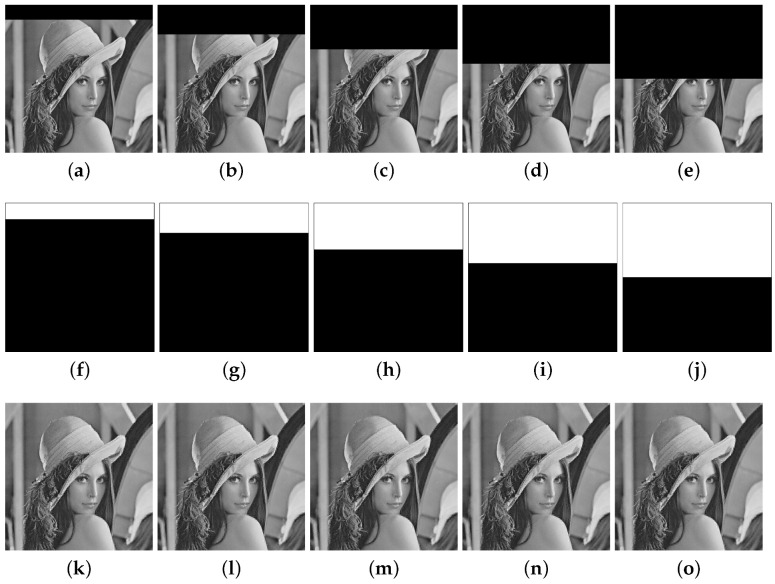
Tampered Lena images: (**a**) 10%, (**b**) 20%, (**c**) 30%, (**d**) 40%, (**e**) 50%. Binary detection images: (**f**) 10%, (**g**) 20%, (**h**) 30%, (**i**) 40%, (**j**) 50%. Recovered images: (**k**) 10%, (**l**) 20%, (**m**) 30%, (**n**) 40%, (**o**) 50%.

The PSNR and SIMM are measures of image degradation caused by the watermark embedding, and the parameter α used in the embedded algorithm modifies the degradation of the original image. Table 1 shows the minimum and maximum values of PSNR and SIMM for several values of α for the 141 original images in the database. It is observed that increasing α (for α>0) slightly decreases the imperceptibility of the watermarked image. Table 2 shows similar results for FPR and FNR for several values of α for the 141 tampered images in the database with tampering rate 50% (the tampered Lena image with this tampering rate is illustrated in Figure 8e). We observe that these performance indicators slightly improve for α>0. Thus, this parameter provides a trade-off among these performance metrics. Hereafter, we fix the value of α to 0.01 in all simulations performed in this section.

Table 3 shows PSNR comparisons between the algorithm Proposed 1 and several existing watermarking fragile methods. It can be seen that the proposed algorithm has better imperceptibility with PSNR higher than 47 dB for the images considered.

Figure 8 shows the tampered Lena images at various tampering rates, the corresponding binary detection images (the detected tampered region is marked in white color, whereas the non-tampered region is in black) and the recovered images. The quality of the recovered image is measured through the ${\mathrm{PSNR}}^{r}$ of the detected tampered region. Table 4 shows a comparison of ${\mathrm{PSNR}}^{r}$ versus tampering rates for several images, where it is seen that the algorithm Proposed 1 provides better recovery performance.

The time (in seconds) required to embed the parity bits into each 512×512 image shown in Figure 7 is in the range [12.15,14.68] (minimum and maximum values), while the range for the extraction of the parity bits is [11.23,13.46]. The algorithms are implemented with the Matlab R2017b program on the Windows 10 Pro operating system running on a personal computer with 3.70 GHz Intel Xeon E5-1620 CPU and 64 GB RAM.

**Figure 9:**
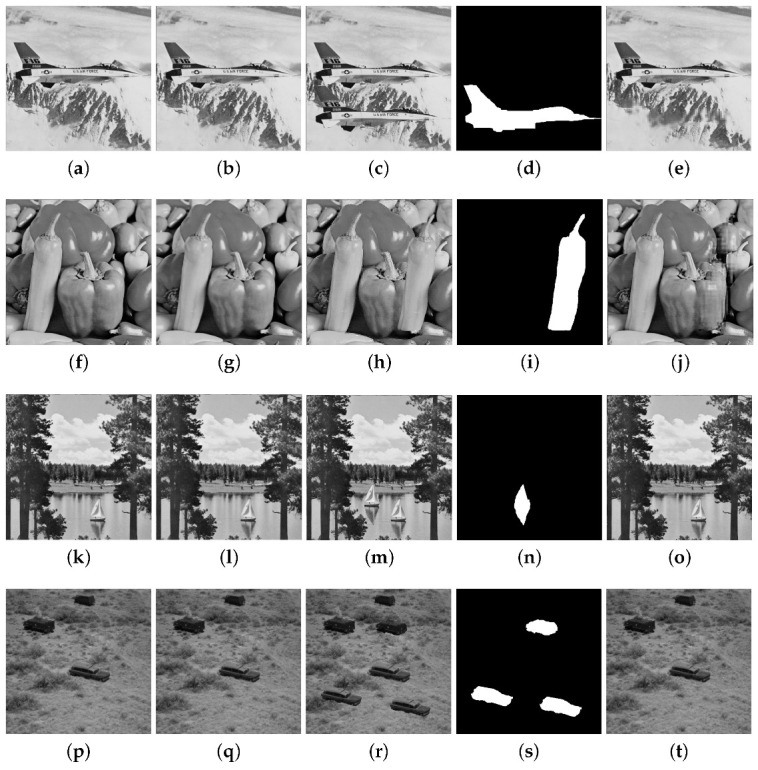
Tampering recovery for the $CA_{1}$ attack: (**a**) original Airplane image, (**b**) watermarked image (PSNR 48.55 dB), (**c**) tampered image (11%), (**d**) binary detection image (FPR = 0.073 and FNR = 0.009), (**e**) recovered image (${\mathrm{PSNR}}^{r}$ = 41.02 dB). (**f**) original Pepper image, (**g**) watermarked image (PSNR = 48.85 dB), (**h**) tampered image (12%), (**i**) binary detection image (FPR = 0.117 and FNR = 0.008), (**j**) recovered image (${\mathrm{PSNR}}^{r}$ = 40.98 dB). (**k**) original Lake image, (**l**) watermarked image (PSNR = 47.95 dB), (**m**) tampered image (2%), (**n**) binary detection image (FPR = 0.100 and FNR = 0.002), (**o**) recovered image (${\mathrm{PSNR}}^{r}$ = 47.52 dB). (**p**) original Countryside image, (**q**) watermarked image (PSNR = 46.13 dB), (**r**) tampered image (6.4%), (**s**) binary detection image (FPR = 0.052 and FNR = 0.007 ), (**t**) recovered image (${\mathrm{PSNR}}^{r}$ = 42.17 dB).

**Figure 10:**
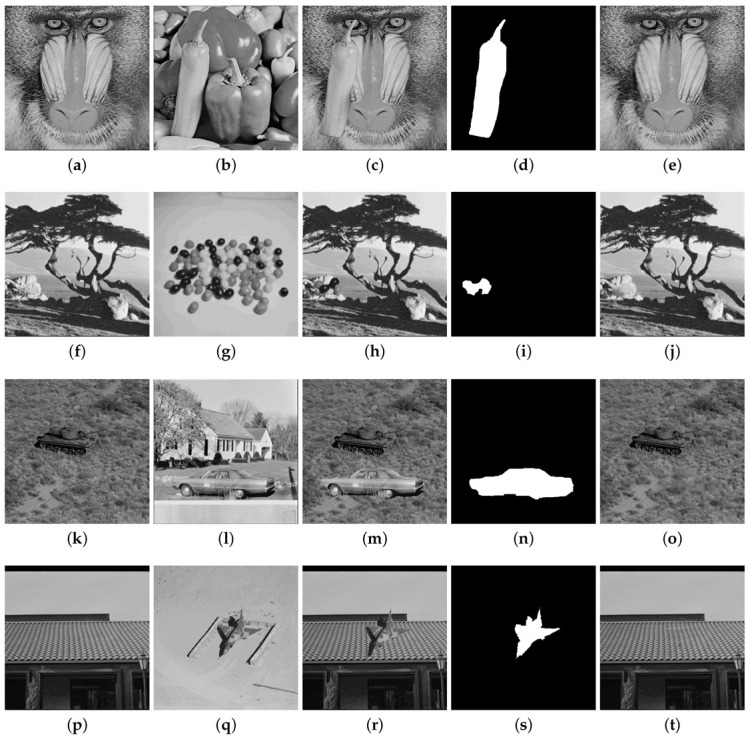
Tampering recovery for the $CA_{2}$ attack: (**a**) watermarked Baboon image (PSNR = 47.51 dB), (**b**) watermarked Pepper image (PSNR = 48.85 dB), (**c**) tampered image (12.2%), (**d**) binary detection image (FPR = 0.099 and FNR = 0.007), (**e**) recovered image (${\mathrm{PSNR}}^{r}$ = 39.86 dB). (**f**) watermarked Tree image (PSNR = 41.15 dB), (**g**) watermarked Seeds image (PSNR = 40.23 dB), (**h**) tampered image (1.40%), (**i**) binary detection image (FPR = 0.087 and FNR = 0.002), (**j**) recovered image (${\mathrm{PSNR}}^{r}$ = 46.32 dB). (**k**) watermarked Tank image (PSNR = 44.33 dB), (**l**) watermarked Car image (PSNR = 42.56 dB), (**m**) tampered image (10.70%), (**n**) binary detection image (FPR = 0.090 and FNR = 0.005), (**o**) recovered image (${\mathrm{PSNR}}^{r}$ = 38.56 dB). (**p**) watermarked Roof image, (**q**) watermarked Airplane image (PSNR = 45.98 dB), (**r**) tampered image (4%), (**s**) binary detection image (FPR = 0.106 and FNR = 0.008), (**t**) recovered image (${\mathrm{PSNR}}^{r}$ = 43.55 dB).

**Figure 11:**
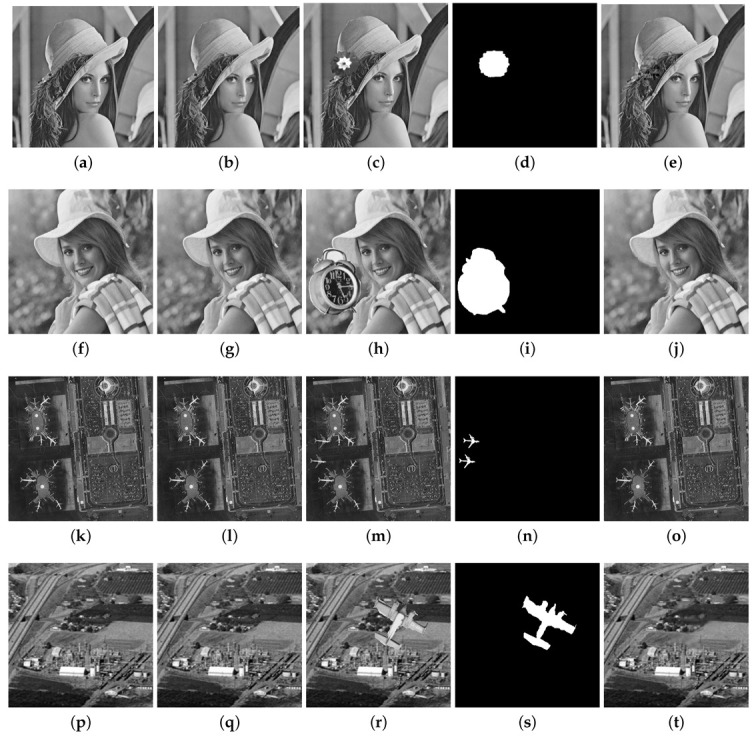
Tampering recovery for normal tampering attack: (**a**) original Lena image, (**b**) watermarked Lena image (PSNR = 49.36 dB), (**c**) tampered image (3%), (**d**) binary detection image (FPR = 0.035 and FNR = 0.003), (**e**) recovered image (${\mathrm{PSNR}}^{r}$ = 47.25 dB). (**f**) original Elaine image, (**g**) watermarked Elaine image (PSNR = 43.36 dB), (**h**) tampered image (12.56%), (**i**) binary detection image (FPR = 0.103 and FNR = 0.008), (**j**) recovered image (${\mathrm{PSNR}}^{r}$ = 40.28 dB). (**k**) original Airport image, (**l**) watermarked image (PSNR = 44.00 dB), (**m**) tampered image (2%), (**n**) binary detection image (FPR = 0.020 and FNR = 0.001), (**o**) recovered image (${\mathrm{PSNR}}^{r}$= 47.42 dB). (**p**) original Aerial View image, (**q**) watermarked image (PSNR = 44.10 dB), (**r**) tampered image (4.8%), (**s**) binary detection image (FPR = 0.135 and FNR = 0.007), (**t**) recovered image (PSNR = 46.12 dB).

The results for the $CA_{1}$ attack for the Airplane, Pepper, Lake and Countryside images are provided in Figure 9. In each row of this figure, the original image, the watermarked image with the PSNR value, the tampered image, the binary detection image with the FPR and FNR values, and the recovered image with the ${\mathrm{PSNR}}^{r}$ value are shown. The PSNR values for these four watermarked images are around 47 dB. The FPR and FNR are, respectively, 0.073 and 0.009 for Airplane, 0.117 and 0.008 for Pepper, 0.100 and 0.002 for Lake, 0.052 and 0.007 for Countryside, and which reveal good tampering detection performance. The Proposed 1 scheme can also achieve good image recovery results with ${\mathrm{PSNR}}^{r}$ around 41 dB for the Airplane, Pepper and Countryside images and around 47 dB for the Lake image. The $CA_{2}$ attack is considered in Figure 10 for the Baboon, Tree, Tank and Roof images. A portion of a watermarked image is copied in another watermarked image, preserving their relative spatial locations. In each row of this figure, two watermarked images with their PSNR values are shown, as well as the tampered image, the binary detection image with the values of FPR and FNR, and the recovered image with the ${\mathrm{PSNR}}^{r}$ value. The PSNR of the watermarked images are higher than 40 dB for these images. The FPR and FNR are respectively 0.099 and 0.007 for Baboon, 0.087 and 0.002 for Tree, 0.090 and 0.005 for Tank, 0.106 and 0.008 for Roof. The recovery results yield ${\mathrm{PSNR}}^{r}$ higher than 38 dB. The normal tampering attack is considered in Figure 11 in which some objects are added to the watermarked images (Lena, Elaine, Airport, and Aerial View).

**Figure 12:**
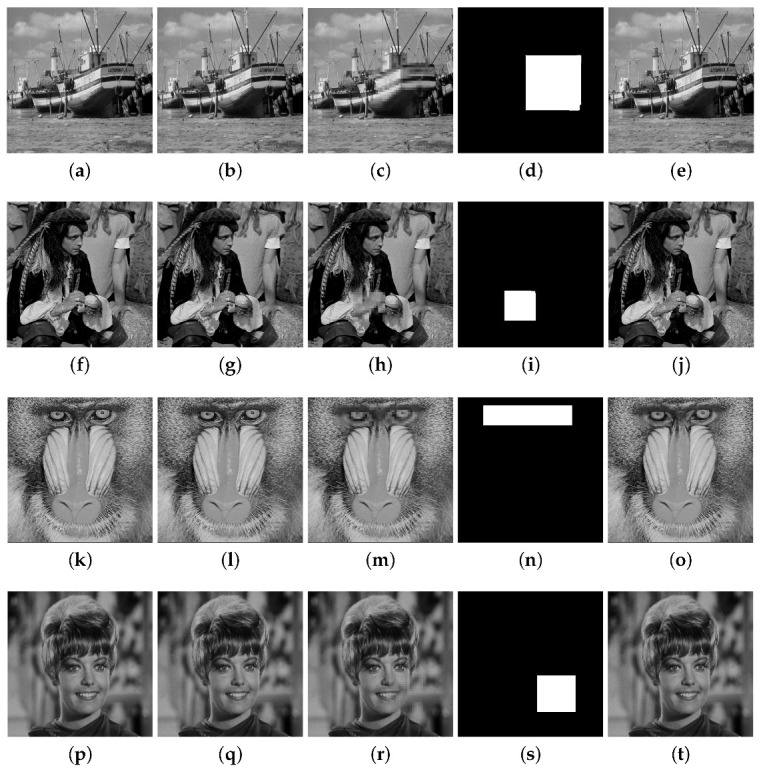
Tampering recovery for the CAA attack: (**a**) original Boat image, (**b**) watermarked image (PSNR = 49.13 dB), (**c**) tampered image (15%), (**d**) binary detection image (FPR = 0.030 and FNR = 0.007), (**e**) recovered image (${\mathrm{PSNR}}^{r}$ = 45.53 dB). (**f**) Sailor original image, (**g**) watermarked image (PSNR = 48.27 dB), (**h**) tampered image (5%), (**i**) binary detection image (FPR = 0.025 and FNR = 0.003), (**j**) recovered image (${\mathrm{PSNR}}^{r}$ = 44.92 dB). (**k**) original Baboon image, (**l**) watermarked image (PSNR = 47.51 dB), (**m**) tampered image (8.5%), (**n**) binary detection image (FPR = 0.026 and FNR = 0.004), (**o**) recovered image (${\mathrm{PSNR}}^{r}$ = 44.31 dB). (**p**) original Zelda image, (**q**) watermarked image (PSNR = 47.04 dB), (**r**) tampered image (7%), (**s**) binary detection image (FPR = 0.012 and FNR = 0.001), (**t**) recovered image (${\mathrm{PSNR}}^{r}$ = 45.18 dB).

**Figure 13:**
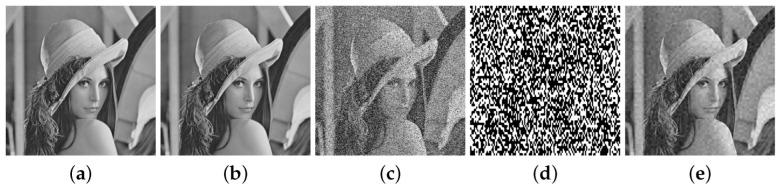
Salt and Pepper attack for the Lena image (**a**) original image, (**b**) watermarked image (PSNR = 49.36 dB), (**c**) tampered image (30%), (**d**) binary detection image (FPR = 0.143 and FNR = 0.084), (**e**) recovered image (${\mathrm{PSNR}}^{r}\;=\;33.78$ dB).

The results for the CAA attack are presented in Figure 12 in which a distortion is created in a certain portion of the watermarked image. The obtained PSNR values are higher than 47 dB for the four images. The FPR and FNR are respectively 0.030, 0.007 for Boat, 0.025, 0.003 for Sailor, 0.026, 0.004 for Baboon, and 0.012, 0.001 for Zelda. The Salt and Pepper attack for the Lena image with d=0.3 is considered in Figure 13. The FPR and FNR are 0.143 and 0.084, respectively.

Some attacks displayed in Figure 9, Figure 10, Figure 12 and Figure 13 have also been considered in the literature. Table 5 compares the ${\mathrm{PSNR}}^{r}$ achieved by the algorithm Proposed 1 and by some existing methods. It is seen that the Proposed 1 technique provides, in some cases, better recovered performance for the considered attacks.

### A BCH Code (31,21,2)

To analyze the impact of the BCH code in the proposed watermarking scheme, we consider the BCH (31,21,2). This code has a greater number of parity bits than the BCH (15,11,1) code, so it is necessary to take into account a greater number of sub-blocks where the parity bits are embedded. The modified algorithm uses the same steps as the previous one, modifying the code used and the level of the DWT, according to the following steps.

1. Repeat this step of the previous algorithm.
2. Apply the one-level DWT decomposition to the original image $C_{O}$. The sub-bands $C_{LH_{1}}$ and $C_{HL_{1}}$ (each one of size M/2×N/2 pixels) are divided into sub-blocks of size 2×2, where the watermarked bits are embedded. The total number of sub-blocks is $\frac{MN}{16}$.
3. Repeat this step of the previous algorithm.
4. Construct the sequence p from the BCH (31,21,2) code as follows. The 21 information bits are obtained by concatenating $k_{1}=2$ bits from $\ell_{4}$ and $k_{2}=19$ from the chaotic map. After scrambling, we obtain $p\;=\;\{p_{1},p_{2},\ldots ,p_{\frac{MN}{64}}\}$, where $p_{i}=p_{i1},p_{i2}$. Each $p_{i}$ is embedded into some sub-blocks of $C_{LH_{1}}$ and $C_{HL_{1}}$.
5. Repeat this step of the previous algorithm. Since there are four times more sub-blocks than subsequences $p_{i}$, after inserting a given $p_{i}$, the next three sub-blocks are not used by the embedded algorithm.
6. Apply the 1-level IDWT and obtain the watermarked image $C_{W}$.

Table 6 shows the PSNR comparison between the algorithm presented in the previous sections (Proposed 1) and the modified one (called Proposed 1-v1). A decrease in the PSNR value is observed due to the insertion at a higher level of the DWT decomposition (1-level). Table 7 presents a ${\mathrm{PSNR}}^{r}$ comparison for several tampering rates, observing a slight increase in the value of ${\mathrm{PSNR}}^{r}$. Table 8 compares the ${\mathrm{PSNR}}^{r}$ for the attacks displayed in Figure 9, Figure 10, Figure 11, Figure 12 and Figure 13. We observe that the modified algorithm presents a better recovery performance for these attacks. This is due to the code modification.

## 6. Performance of the Proposed Algorithm for Colored Images

**Figure 14:**
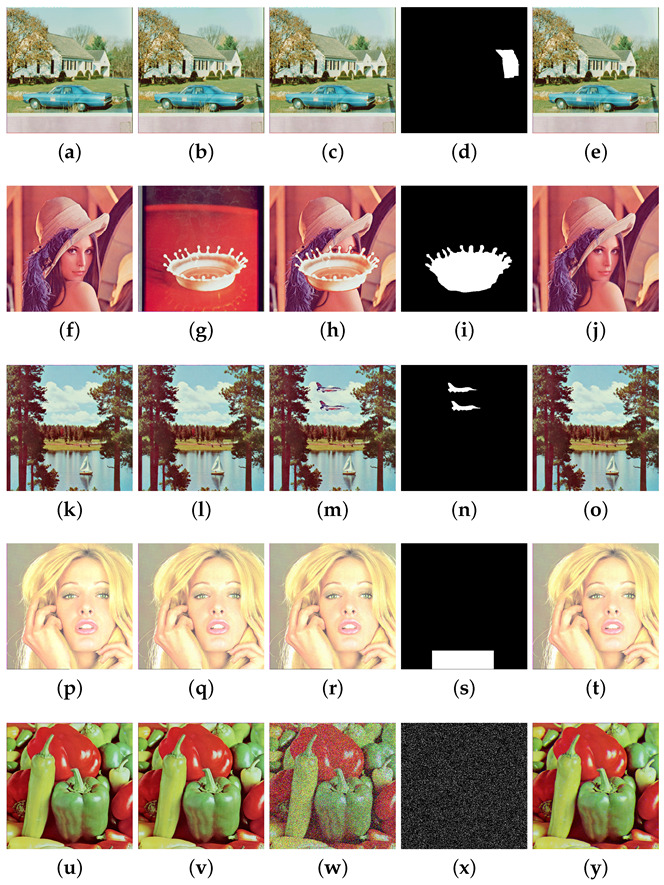
Different attacks on colored images (a-e) tampering recovery for the CA1 attack (**a**) original Car image, (**b**) watermarked Car image (PSNR = 47.35 dB), (**c**) tampered image (3%), (**d**) binary detection image (FPR = 0.069 and FNR = 0.008), (**e**) recovered image (${\mathrm{PSNR}}^{r}$ = 51.61 dB), (**f**–**j**) tampering recovery for the $CA_{2}$ attack: (**f**) watermarked Lena image (PSNR = 47.26 dB), (**g**) watermarked Splash image (PSNR = 46.75 dB), (**h**) tampered image (16%), (**i**) binary detection image (FPR = 0.083 and FNR = 0.011), (**j**) recovered image (${\mathrm{PSNR}}^{r}$ = 47.56 dB), (**k**–**o**) tampering recovery for normal tampering attack: (**k**) original Lake image, (**l**) watermarked Lake image (PSNR = 46.42 dB), (**m**) tampered image (1.5%), (**n**) binary detection image (FPR = 0.051 and FNR = 0.005), (**o**) recovered Tiffany image (${\mathrm{PSNR}}^{r}$ = 49.34 dB), (**p**–**t**) tampering recovery for the CAA attack: (**p**) original Tiffany image, (**q**) watermarked image (PSNR = 47.25 dB), (**r**) tampered image (7%), (**s**) binary detection image (FPR = 0.023 and FNR = 0.004), (**t**) recovered image (${\mathrm{PSNR}}^{r}$ = 51.04 dB), (**u**–**y**) Salt and Pepper attack (**u**) original Pepper image, (**v**) watermarked Pepper image PSNR = 48.01 dB, (**w**) tampered image (30%), (**x**) binary detection image (FPR = 0.103 and FNR = 0.062), (**y**) recovered image (${\mathrm{PSNR}}^{r}$ = 34.08 dB).

The performance of the proposed algorithms in colored images is analyzed in terms of imperceptibility, detection and recovery. We also present comparisons with literature results. The original colored image $C_{O}$ is represented by three components R, G, and B, each one of size 512×512. A fragile watermarking algorithm in a grayscale image is applied in each component. We adopt the same performance metrics used for grayscale images. Table 9 presents an imperceptibility comparison between the proposed algorithms and some existing ones for several images. All proposed algorithms present better imperceptibility results, and the highest PSNR values are achieved by Proposed 1. Table 10 shows a comparison of ${\mathrm{PSNR}}^{r}$ versus several tampered rates. Some attacks presented in the previous chapter are presented in Figure 14 with recovered results for the algorithm Proposed 1. Behavior similar to that obtained with grayscale images is observed, obtaining FNR and FPR values close to zero (desired values) and ${\mathrm{PSNR}}^{r}$ values higher than 34 dB.

## 7. Conclusions

This article presented a new self-embedding fragile watermarking algorithm for tamper detection and content recovery in images. The watermarked bits are the parity bits of a BCH code, in which its information sequence is composed of chaotic bits and bits obtained from the original image. The watermarked bits are embedded in the original image in the frequency domain using the DWT. The parameter α establishes a trade-off between imperceptibility and recovery. After investigating the trade-off between the imperceptibility, detection of tampered areas, and recovery capability of the algorithm, we compare its performance with that of some existing schemes. We conclude that the algorithm is competitive in terms of several metrics, such as PSNR, SIMM, FPR, FNR, and ${\mathrm{PSNR}}^{r}$. The joint application of chaotic bits and BCH codes not only contributes to the recovery of the image information in the tampered areas, but also provides security, and the existence of a greater number of parity bits leads to higher recoverability. A natural continuation of this work is the incorporation of codes with unequal error protection, since part of the information bits is known at the extraction algorithm. Another topic for future research is to consider chaotic maps with high nonlinearities and constant chaos for a wide parameter range [51]. 

## Figures and Tables

**Table 1:** Minimum and maximum PSNR and SIMM for several values of α for the 141 images from the USC-SIPI database.

Metrics	α=0		α=0.01		α=0.5		α=1		α=2	
	Min	Max	Min	Max	Min	Max	Min	Max	Min	Max
PSNR	∞	∞	37.46	51.04	37.37	50.98	37.34	50.92	37.32	50.80
SSIM	1	1	0.953	0.994	0.953	0.994	0.953	0.994	0.952	0.990

**Table 2:** Minimum and maximum FPR and FNR for several values of α for the 141 images with tampering rate 50%.

Metrics	α=0		α=0.01		α=0.5		α=1		α=2	
	Min	Max	Min	Max	Min	Max	Min	Max	Min	Max
FPR	0.438	0.662	0.105	0.201	0.101	0.195	0.099	0.194	0.098	0.194
FNR	0.124	0.305	0	0.021	0	0.019	0	0.018	0	0.016

**Table 3:** PSNR comparison for several original images.

Scheme	PSNR					
	Lena	Airplane	Boat	Lake	Pepper	Baboon
Proposed 1	49.36	48.55	49.13	47.95	48.85	47.51
[12]	44.27	43.85	44.37	42.49	44.23	44.31
[14]	44.14	44.14	44.28	44.19	44.17	44.01
[17]	41.00	47.33	48.02	47.11	47.23	47.29
[15]	38.77	39.03	38.67	38.28	37.99	38.49
[22]	45.82	45.81	45.76	45.79	45.80	45.79
[23]	44.32	44.74	45.06	44.73	44.57	45.11
[20]	42.11	41.38	41.49	42.77	42.12	42.23

**Table 4:** ${\mathrm{PSNR}}^{r}$ versus tampered rate comparison for several original images.

Image	Scheme	Tampered Rate %				
		10	20	30	40	50
Lena	Proposed 1	51.35	48.78	47.18	45.52	43.12
	[22]	44.16	41.84	40.22	38.17	36.55
	[23]	40.52	37.60	35.89	31.92	29.32
	[17]	49.47	44.39	41.23	38.58	36.61
Baboon	Proposed 1	50.90	48.25	46.82	45.80	42.93
	[22]	41.18	42.58	41.03	38.45	34.82
	[23]	41.80	39.75	36.16	32.51	30.80
	[17]	38.69	35.55	33.95	32.93	32.13
Peppers	Proposed 1	51.08	48.80	46.88	45.07	43.05
	[22]	44.07	41.74	40.39	39.19	38.02
	[23]	41.35	39.60	35.97	33.05	31.68
	[17]	42.84	40.54	38.32	36.76	35.17
Airplane	Proposed 1	50.84	48.93	47.01	45.33	43.07
	[22]	41.99	40.24	38.57	36.99	35.95
	[23]	40.38	38.20	36.07	33.94	31.91
	[17]	46.59	44.54	42.83	40.32	36.79

**Table 5:** ${\mathrm{PSNR}}^{r}$ achieved by the proposed algorithm and by some existing methods.

Figure	Image	${\mathrm{PSNR}}^{r}$		
		Proposed 1	Other Schemes	
Figure 9j	Pepper	40.98	[36]	33.59
Figure 9o	Lake	47.52	[20]	33.82
Figure 10e	Baboon	39.86	[20]	30.33
Figure 11o	Airport	47.42	[34]	46.03
Figure 12e	Boat	45.53	[14]	35.41
Figure 13e	Lena	33.78	[17]	40.68

**Table 6:** PSNR comparison for several original images.

Scheme	PSNR					
	Lena	Airplane	Boat	Lake	Pepper	Baboon
Proposed 1	49.36	48.55	49.13	47.95	48.85	47.51
Proposed 1-v1	47.52	46.28	47.07	45.67	46.28	45.21

**Table 7:** ${\mathrm{PSNR}}^{r}$ versus tampered rate comparison for several original images.

Image	Scheme	Tampered Rate %				
		10	20	30	40	50
Lena	Proposed 1	51.35	48.78	47.18	45.52	43.12
	Proposed 1-v1	52.38	49.80	48.52	46.10	44.00
Baboon	Proposed 1	50.90	48.25	46.82	45.80	42.93
	Proposed 1-v1	51.00	48.42	47.01	48.92	43.01
Peppers	Proposed 1	51.08	48.80	46.88	45.07	43.05
	Proposed 1-v1	51.19	48.95	47.01	45.19	43.18
Airplane	Proposed 1	50.84	48.93	47.01	45.33	43.07
	Proposed 1-v1	50.96	49.07	47.13	45.49	43.16

**Table 8:** ${\mathrm{PSNR}}^{r}$ comparison for several attacks of proposed algorithms.

Figure	Image	${\mathrm{PSNR}}^{r}$	
		Proposed 1	Proposed 1-v1
Figure 9j	Peppers	40.98	43.16
Figure 9o	Lake	47.52	49.83
Figure 10e	Baboon	39.86	42.07
Figure 11o	Airport	47.42	49.28
Figure 12e	Boat	45.53	46.90
Figure 13e	Lena	33.78	35.21

**Table 9:** PSNR comparison for several original colored images.

Scheme	PSNR					
	Lena	Airplane	House	Sailboat	Pepper	Baboon
Proposed 1	51.26	51.27	51.07	51.10	51.21	50.98
Proposed 1-v1	51.08	51.11	50.98	51.08	51.13	50.70
[22]	46.45	46.23	46.22	46.18	46.03	46.24
[16]	44.60	44.69	44.66	44.61	44.54	44.64
[19]	46.37	48.32	46.23	47.12	46.3	46.17

**Table 10:** ${\mathrm{PSNR}}^{r}$ versus tampered rate comparison for several color original images.

Image	Scheme	Tampered Rate %				
		10	20	30	40	50
Lena	Proposed 1	52.08	49.13	48.02	46.13	44.28
	Proposed 1-v1	52.31	49.82	48.53	46.68	45.04
	[22]	44.22	39.77	37.64	35.91	34.80
	[16]	37.16	33.83	31.48	29.07	26.96
	[33]	49.47	44.39	41.23	38.58	36.61
Baboon	Proposed 1	51.23	48.97	47.86	46.20	44.40
	Proposed 1-v1	51.76	49.28	48.61	47.55	45.88
	[22]	42.00	38.05	37.05	35.00	32.50
	[16]	35.85	31.87	28.38	25.59	23.59
	[33]	29.50	26.77	24.98	22.99	21.66
Peppers	Proposed 1	52.00	48.92	47.90	46.77	44.16
	Proposed 1v-1	53.60	49.71	48.96	47.90	45.73
	[22]	44.02	40.00	39.20	37.00	35.92
	[16]	37.38	34.63	32.48	29.89	27.31
	[33]	35.67	32.36	30.07	28.62	27.24
Airplane	Proposed 1	51.72	49.28	47.91	46.60	44.17
	Proposed 1-v1	52.66	50.93	49.08	47.95	45.78
	[22]	41.90	40.00	39.00	36.95	35.00
	[16]	36.51	33.40	31.28	28.51	25.99
	[33]	42.72	34.81	30.24	28.16	26.42

## Data Availability

Not applicable.

## References

[1] Naskar R., Chakraborty R.S. (2014). Reversible Digital Watermarking: Theory and Practices.

[2] Rakhmawati L., Wirawan W., Suwadi S. (2019). A recent survey of self-embedding fragile watermarking scheme for image authentication with recovery capability. EURASIP J. Image Video Process..

[3] Gul E., Ozturk S. (2021). A novel pixel-wise authentication-based self-embedding fragile watermarking method. Multimed. Syst..

[4] Kosuru D., Swain G., Kumar N., Pradhan A. (2022). Image tamper detection and correction using Merkle tree and remainder value differencing. Optik.

[5] Shih F.Y. (2010). Image Processing and Pattern Recognition: Fundamentals and Techniques.

[6] Moosazadeh M., Ekbatanifard G. (2019). A new DCT-based robust image watermarking method using teaching-learning-Based optimization. J. Inf. Secur. Appl..

[7] Ko H.J., Huang C.T., Horng G., Wang S.J. (2020). Robust and blind image watermarking in DCT domain using inter-block coefficient correlation. Inf. Sci..

[8] Zhou X., Ma Y., Mohammed M.A., Damaševičius R. (2021). A reversible watermarking system for medical colored images: Balancing capacity, imperceptibility, and robustness. Electronics.

[9] Gul E., Toprak A.N. (2023). Contourlet and discrete cosine transform based quality guaranteed robust image watermarking method using artificial bee colony algorithm. Expert Syst. Appl..

[10] Peng Y., Niu X., Fu L., Yin Z. (2018). Image authentication scheme based on reversible fragile watermarking with two images. J. Inf. Secur. Appl..

[11] Qin C., Wang H., Zhang X., Sun X. (2016). Self-embedding fragile watermarking based on reference-data interleaving and adaptive selection of embedding mode. Inf. Sci..

[12] Qin C., Ji P., Zhang X., Dong J., Wang J. (2017). Fragile Image Watermarking With Pixel-wise Recovery Based on Overlapping Embedding Strategy. Signal Process..

[13] Sreenivas K., Kamakshiprasad V. (2017). Improved image tamper localisation using chaotic maps and self-recovery. J. Vis. Commun. Image Represent..

[14] Tai W., Liao Z. (2018). Image self-recovery with watermark self-embedding. Signal Process. Image Commun..

[15] Abdelhakim A., Saleh H., Abdelhakim M. (2019). Fragile watermarking for image tamper detection and localization with effective recovery capability using K-means clustering. Multimed. Tools Appl..

[16] Molina J., Garcia B., Ponomaryov V., Reyes R., Sadovnychiy S., Cruz C. (2020). An effective fragile watermarking scheme for colored image tampering detection and self-recovery. Signal Process. Image Commun..

[17] Lee C., Shen J., Chen Z., Agrawal S. (2019). Self-Embedding authentication watermarking with effective tampered location detection and high-quality image recovery. Sensors.

[18] Sarreshtedari S., Akhaee M.A., Abbasfar A. (2018). Source channel coding-based watermarking for self-embedding of JPEG images. Signal Process. Image Commun..

[19] Al-Otum H.M., Ellubani A.A.A. (2022). Secure and effective colored image tampering detection and self restoration using a dual watermarking approach. Optik.

[20] Wu H.C., Fan W.L., Tsai C.S., Ying J.J.C. (2022). An image authentication and recovery system based on discrete wavelet transform and convolutional neural networks. Multimed. Tools Appl..

[21] Klington A.G., Ramesh K., Kadry S. (2021). Cost-Effective watermarking scheme for authentication of digital fundus images in healthcare data management. Inf. Technol. Control.

[22] Bolourian Haghighi B., Taherinia A.H., Mohajerzadeh A.H. (2019). TRLG: Fragile blind quad watermarking for image tamper detection and recovery by providing compact digests with optimized quality using LWT and GA. Inf. Sci..

[23] Jafari Barani M., Yousefi Valandar M., Ayubi P. (2019). A new digital image tamper detection algorithm based on integer wavelet transform and secured by encrypted authentication sequence with 3D quantum map. Optik.

[24] Azeroual A., Afdel K. (2017). Real-time image tamper localization based on fragile watermarking and Faber-Schauder wavelet. AEU-Int. J. Electron. Commun..

[25] Raj N.N., Shreelekshmi R. (2022). Fragile watermarking scheme for tamper localization in images using logistic map and singular value decomposition. J. Vis. Commun. Image Represent..

[26] Rawat S., Raman B. (2011). A chaotic system based fragile watermarking scheme for image tamper detection. AEU-Int. J. Electron. Commun..

[27] Li M., Xiao D., Liu H., Bai S. (2016). A recoverable chaos-based fragile watermarking with high PSNR preservation. Secur. Commun. Netw..

[28] Lefévre P., Carré P., Gaborit P. (2019). Application of rank metric codes in digital image watermarking. Signal Process. Image Commun..

[29] Fan M., Wang H. (2018). An enhanced fragile watermarking scheme to digital image protection and self-recovery. Signal Process. Image Commun..

[30] Qin C., Ji P., Wang J., Chang C.C. (2017). Fragile image watermarking scheme based on VQ index sharing and self-embedding. Multimed. Tools Appl..

[31] Hsu C.S., Tu S.F. (2016). Image tamper detection and recovery using adaptive embedding rules. Measurement.

[32] Singh D., Singh S.K. (2016). Effective self-embedding watermarking scheme for image tampered detection and localization with recovery capability. J. Vis. Commun. Image Represent..

[33] Sinhal R., Ansari I.A., Ahn C.W. (2020). Blind image watermarking for localization and restoration of color images. IEEE Access.

[34] Yuan X., Li X., Liu T. (2021). Gauss Jordan elimination-based image tampering detection and self-recovery. Signal Process. Image Commun..

[35] Rezaei M., Taheri H. (2022). Digital image self-recovery using CNN networks. Optik.

[36] Rajput V., Ansari1 I. (2020). Image tamper detection and self-recovery using multiple median watermarking. Multimed. Tools Appl..

[37] Tan Y., Jiaohua Q. (2019). A robust watermarking scheme in YCbCr color space based on channel coding. IEEE Access.

[38] Lefévre P., Carré P., Fontaine C., Gaborit P., Huang J. (2022). Efficient image tampering localization using semi-fragile watermarking and error control codes. Signal Process..

[39] Lin S., Costello D.J. (2004). Error Control Coding.

[40] Strogatz S.H. (2001). Nonlinear Dynamics and Chaos.

[41] Lan R., He J., Wang S., Liu Y., Luo X. (2019). A parameter-selection-based chaotic system. IEEE Trans. Circuits Syst. Ii Express Briefs.

[42] Zhou Y., Hua Z., Pun C.M., Philip Chen C.L. (2015). Cascade chaotic system with applications. IEEE Trans. Cybern..

[43] Callegari S., Fabbri M., Beirami A. (2016). Very low cost chaos-based entropy source for the retrofit or design augmentation of networked devices. Analog. Integr. Circuits Signal Process..

[44] Atawneh S., Almomani A., Al Bazar H., Sumari P., Gupta B. (2017). Secure and imperceptible digital image steganographic algorithm based on diamond encoding in DWT domain. Multimed. Tools Appl..

[45] Gangadhar Y., Giridhar Akula V.S., Reddy P.C. (2018). An evolutionary programming approach for securing medical images using watermarking scheme in invariant discrete wavelet transformation. Biomed. Signal Process. Control.

[46] Farghaly S.H., Ismail S.M. (2020). Floating-point discrete wavelet transform-based image compression on FPGA. AEU-Int. J. Electron. Commun..

[47] Al-Shayea T.K., Mavromoustakis C.X., Batalla J.M., Mastorakis G. (2019). A hybridized methodology of different wavelet transformations targeting medical images in IoT infrastructure. Measurement.

[48] El-Hoseny H.M., El Kareh Z.Z., Mohamed W.A., El Banby G.M., Mahmoud K.R., Faragallah O.S., El-Rabaie S., El-Madbouly E., Abd El-Samie F.E. (2019). An optimal wavelet-based multi-modality medical image fusion approach based on modified central force optimization and histogram matching. Multimed. Tools Appl..

[49] Thakkar F.N., Srivastava V.K. (2017). A fast watermarking algorithm with enhanced security using compressive sensing and principle components and its performance analysis against a set of standard attacks. Multimed. Tools Appl..

[50] Stephane M. (2009). A Wavelet Tour of Signal Processing.

[51] Hua Z., Zhou B., Zhou Y. (2019). Sine chaotification model for enhancing chaos and its hardware implementation. IEEE Trans. Ind. Electron..

